# Irreversibility minimization analysis of ferromagnetic Oldroyd-B nanofluid flow under the influence of a magnetic dipole

**DOI:** 10.1038/s41598-021-84254-1

**Published:** 2021-02-26

**Authors:** Muhammad Ramzan, Fares Howari, Jae Dong Chung, Seifedine Kadry, Yu-Ming Chu

**Affiliations:** 1 Department of Computer Science, Bahria University, Islamabad, 44000 Pakistan; 2 Department of Mechanical Engineering, Sejong University, Seoul, 143-747 Korea; 3 College of Natural and Health Sciences, Zayed University, 144543 Abu Dhabi, UAE; 4 Department of Mathematics and Computer Science, Faculty of Science, Beirut Arab University, Beirut, 115020 Lebanon; 5 Department of Mathematics, Huzhou University, Huzhou, 313000 People’s Republic of China; 6 Hunan Provincial Key Laboratory of Mathematical Modeling and Analysis in Engineering, Changsha University of Science and Technology, Changsha, 410114 People’s Republic of China

**Keywords:** Software, Mechanical engineering

## Abstract

Studies highlighting nanoparticles suspensions and flow attributes in the context of their application are the subject of current research. In particular, the utilization of these materials in biomedical rheological models has gained great attention. Magneto nanoparticles have a decisive role in the ferrofluid flows to regulate their viscoelastic physiognomies. Having such substantial interest in the flow of ferrofluids our objective is to elaborate the melting heat transfer impact in a stretched Oldroyd-B flow owing to a magnetic dipole in the presence of entropy generation optimization. Buongiorno nanofluid model expounding thermophoretic and Brownian features are considered. Moreover, activation energy with chemical reaction is also considered. The Cattaneo–Christov heat flux model is affianced instead of conventional Fourier law. The renowned bvp4c function of MATLAB is utilized to handle the nonlinearity of the system. Impacts of miscellaneous parameters are portrayed through graphical fallouts and numeric statistics. Results divulge that the velocity and temperature profiles show the opposite trend for growing estimates of the ferromagnetic parameter. It is also noticed that the temperature ratio parameter diminishes the entropy profile. Moreover, it is seen that the concentration profile displays a dwindling trend for the Brownian motion parameter and the opposite trend is witnessed for the thermophoretic parameter.

## Introduction

Fluids used in industries are mostly non-Newtonian ^1–7^. Stress and deformation rates have a nonlinear relationship in these liquids. Examples like animal blood, molten polymers, alcoholic beverages, etc. may be quoted for non-Newtonian fluids. These fluids are generally classified into three basic classes; the rate type; the integral type; and the differential type. Owing to simplification in mathematical modeling of differential type fluids many researchers have taken interest in their related problems. In Differential type models the shear stress is expressed in the form of the velocity components. Nevertheless, fewer efforts are seen in the case of rate type fluids. One of the renowned rate type fluid models named Maxwell fluid model with only relaxation time information possesses a limited scope. But the Oldroyd-B fluid model ^8^ provides both properties of relaxation and retardation times characteristics. Researchers have shown immense curiosity to explore the numerous aspects of this important non-Newtonian fluid. The Oldroyd-B nanofluid flow with thermal and solutal stratifications in the presence of nonlinear thermal radiation aspects and chemical reaction in the vicinity of a stagnation point is analyzed by Irfan et al. ^9^. It is noticed in this study that the temperature and concentration of the fluid are declined for thermal and solutal stratifications. The flow of the Oldroyd-B fluid owing to a rotating disk near a stagnation point with nonlinear thermal radiation is examined numerically by Hafeez et al. ^10^. The salient outcome of this exploration is that the rate of heat flux deteriorates for the Brownian and thermophoretic parameters near the wall. A similar trend is near the wall is witnessed in the concentration gradient for the mass transfer parameter. Khan et al. ^11^ deliberated the Oldroyd-B nanofluid bioconvection flow over an oscillatory extended surface with motile microorganisms using an effective Prandtl approach. It is comprehended here that the motile microorganism’s profile is diminished for varied estimates of bioconvected Peclet number. The convective flow of Oldroyd-B fluid in the vicinity of a stagnation point with autocatalytic chemical reaction and ohmic heating over an extended surface is examined analytically by Wang et al. ^12^. The major upshot of the envisioned problem is that the heat transfer rate is dominant in the case of the Biot number. It is also inferred from this study that both autocatalytic reactions show the opposite behavior for the concentration of the fluid. The multiple fractional solutions of the Oldroyd-B bio-nanofluid flow with variable velocity and ramped wall heating are studied by Saqib et al. ^13^. Khan et al. ^14^ investigated the flow of Oldroyd-B nanofluid flow past an exponentially convectively heated stretched surface. The stability of the nanofluid flow is strengthened by adding the gyrotactic microorganisms. The major outcome of the presented model is that the fluid velocity is hindered for high relaxation parameter estimates but an opposing behavior is observed for retardation time. However, the fluid temperature is escalated for the relaxation parameter, and a conflicting trend is seen for the retardation parameter. The numerical analysis of the Oldroyd-B nanofluid flow over a rotating disk with the radiative impacts and Arrhenius activation energy is done by Waqas et al. ^15^. The stability of the fluid is augmented by the addition of microorganisms. It is comprehended that both radial and azimuthal velocities are on the decline when the rotation of the disk is improved.

The Fourier law has been well known for measuring the heat transfer rate for the last two centuries but with a limitation labeled “Paradox of heat conduction”. Cattaneo ^16^ found a remedy to this limitation by introducing thermal relaxation time which resolves the inconsistency in heat conduction. Christov ^17^ then improved the model by swapping Maxwell–Cattaneo model time derivative by Oldroyd upper-convected derivative. This modified model is named as “Cattaneo–Christov heat flux model”. Ciarletta and Straughan ^18^ have substantiated the uniqueness of the solutions of time-dependent problems, attained from Cattaneo–Christov equations. Ramzan et al. ^19^ deliberated the MHD third-grade fluid flow with homogeneous heterogeneous reactions and the modified Fourier law of heat flux accompanying convective boundary condition passed through a linearly extended sheet. Lu et al. ^20^ considered the rotating flow of 3D MHD Maxwell fluid with activation energy and non-Fourier heat flux. The numerical simulation of the convective boundary of hydromagnetic stagnation point flow with non-Newtonian Williamson liquid with impacts of the Cattaneo–Christov model over an extended surface is executed by Ramzan et al. ^21^. Some recent investigations featuring the Cattaneo–Christov heat effect may be found in ^22–27^.

The aspect of magneto-hydrodynamic principles to change the flow field by modifying the boundary layer structure has established the importance of MHD flow in various industrial applications. The MHD is being valued by fluid dynamists, physiologists and medical specialists reasoned to its applicability in bio-medical engineering and overcoming a variety of pathological issues. Ramzan et al. ^28^ debated the impact of Newtonian heating on the MHD couple stress fluid flow over an extended sheet by considering the Joule heating and viscous dissipation. Seth et al. ^29^ investigated the time-independent MHD Casson fluid flow influenced by Newtonian heating, Joule heating, thermo-diffusion, and viscous dissipation along with a vertical moving plate over a non-Darcy porous media.

Nanofluid is an amalgamation of suspended metallic nanoparticles and some base fluid say water. The thermal performance of the base fluid is dramatically enhanced with the insertion of metallic nanoparticles. The application of nanofluids has revolutionized modern engineering processes especially in manufacturing small gadgets. The nanofluid has gained great importance in the field of nanotechnology as literature shows the attempts made by scientists and researchers to discover the various aspects of nanofluids. Das et al. ^30^ through their pioneered work discussed nanofluids, which are a blend of nano-sized metallic particles and base fluid. The heat transfer rate is much larger in nanofluids in comparison to conventional base fluid. In fluid mechanics, the flow of magneto nanofluids is an immensely important study area due to vital applications such as drug release, synergistic effects, asthma treatment, and riddance of tumors with hyperthermia. The MHD second-grade nonliquid flow through a bi-directional stretching sheet induced by Cattaneo–Christov concentration and thermal diffusion flux is analyzed by Ramzan et al. ^31^. Safwa Khashi’ie et al. ^32^ dealt with the magneto nanofluid flow through an absorbent stretching/shrinking surface with the impact of the dual stratification phenomenon. Moreover, the problem equation is also accompanied by the Cattaneo–Christov heat flux and Buongiorno model. Various studies on MHD nanofluids with different models can be seen in the literature ^33–41^.

Ferrofluids are categorized as a class of magnetizable liquids with interesting characteristics having a significant influence on technology. The single-domain magnetic particles usually measuring 10 nm, dispersed in a carrier liquid forming a colloidal suspension is known as ferrofluid. The worth mentioning industrial applications of these fluids are in nuclear power plants, laser, aerodynamics, semiconductor processing, filtration, avionics, crystal processing, robotics, refrigeration, drawing plastic, and computer peripherals, etc. This wide-ranging beneficial applicability has compelled scientists and researchers to accelerate the research on these fluids. Initially, the ferrofluid stagnation point flow with the influence of magnetic dipole by the heated sheet is scrutinized by Neuringer ^42^. The ferrofluid with a magnetic dipole is studied by Andersson and Valnes ^43^. Kefayati ^44^ addressed the features in ferromagnetic fluid with a heated cavity by executing the Lattice Boltzmann methods (LBM) technique. Waqas et al. ^45^ examined the Carreau ferrofluid flow by considering the Buongiorno model in a moving stretchable sheet with the magnetic dipole. Furthermore, the consequences of nonlinear radiation and heat generation were taken into account. Ijaz and Ayub ^46^ investigated that the radiative ferromagnetic Maxwell fluid flow in a stratified medium and chemically reaction through a preamble stretchable sheet. Hayat et al. ^47^ scrutinized the flow of 2D ferromagnetic Williamson liquid past an extended sheet with a magnetic dipole, viscous dissipation, and thermal radiation. Furthermore, some recent studies elaborating ferromagnetic fluid with magnetic dipole are discussed through the refs. ^48–54^.

The importance of the second law of thermodynamics in the thermodynamic systems is dominant in comparison to the first law. This leads to the concept of entropy generation that directly affects the performance of a system. Entropy is the absence of the energy in some systems for effective mechanical work that considers as an outcome of disorder and uncertainty in liquid molecules. Scientists and researchers have focused on entropy generation owing to its prominence in mechanical systems. Bejan's ^55^ coined work introduces the concept of entropy generation. Khan et al. ^56^ analyzed analytically the flow of Carreau nanofluid flow with entropy generation over an extended surface. They also amalgamated the effects of thermal radiation, viscous and ohmic dissipations with heat source/sink. Their observations reveal that entropy generation is exaggerated for mounting estimates of the magnetic parameter. The flow of MHD Carreau nanofluid with entropy generation optimization and thermal radiation over a shrinking surface is deliberated numerically by Bhatti et al. ^57^. They concluded that the thermal radiation parameter and the Prandtl number portray conflicting behavior on the temperature profile. Kefayati and Tang ^58^ studied the entropy generation of Carreau fluid with thermal and solutal natural convection with MHD in a heated enclosure with two clod inner circular cylinders numerically employing the Lattice Boltzmann technique. They noticed that Rayleigh's number boosts the entropy generations and Hartmann's number lowers the total entropy generation.

The aforesaid studies review discloses that the flow of Oldroyd-B nanofluid is discussed with numerous geometries by various authors. Nevertheless, no one has discussed the Oldroyd-B nanofluid flow with magnetic dipole influence and modified Fourier law in the literature. Thus, our objective is to examine the Oldroyd-B nanofluid flow with magnetic dipole influence and modified Fourier law. The activation energy with melting heat transfer effects at the surface of the sheet is also taken into account. The assumed model will also expound on the shear thinning and thickening traits by considering the Oldroyd-B fluid model. The numerical solution for the nonlinear system is attained and results are given in the form of varied graphs and numerically erected tabulated estimates. While evaluating this investigation, the objective of the present study is to answer the following questions:

i. What is the impact of the ferromagnetic on fluid velocity and temperature?
ii. How Brownian motion and thermophoretic effect influences the fluid concentration?
iii. What is the role of entropy in this model?
iv. How Cattaneo–Christov heat flux affects the nanofluid flow?
v. Impact of the melting heat on the nanofluid flow?

## Model configuration, assumptions, and governing equations

**Figure 1:**
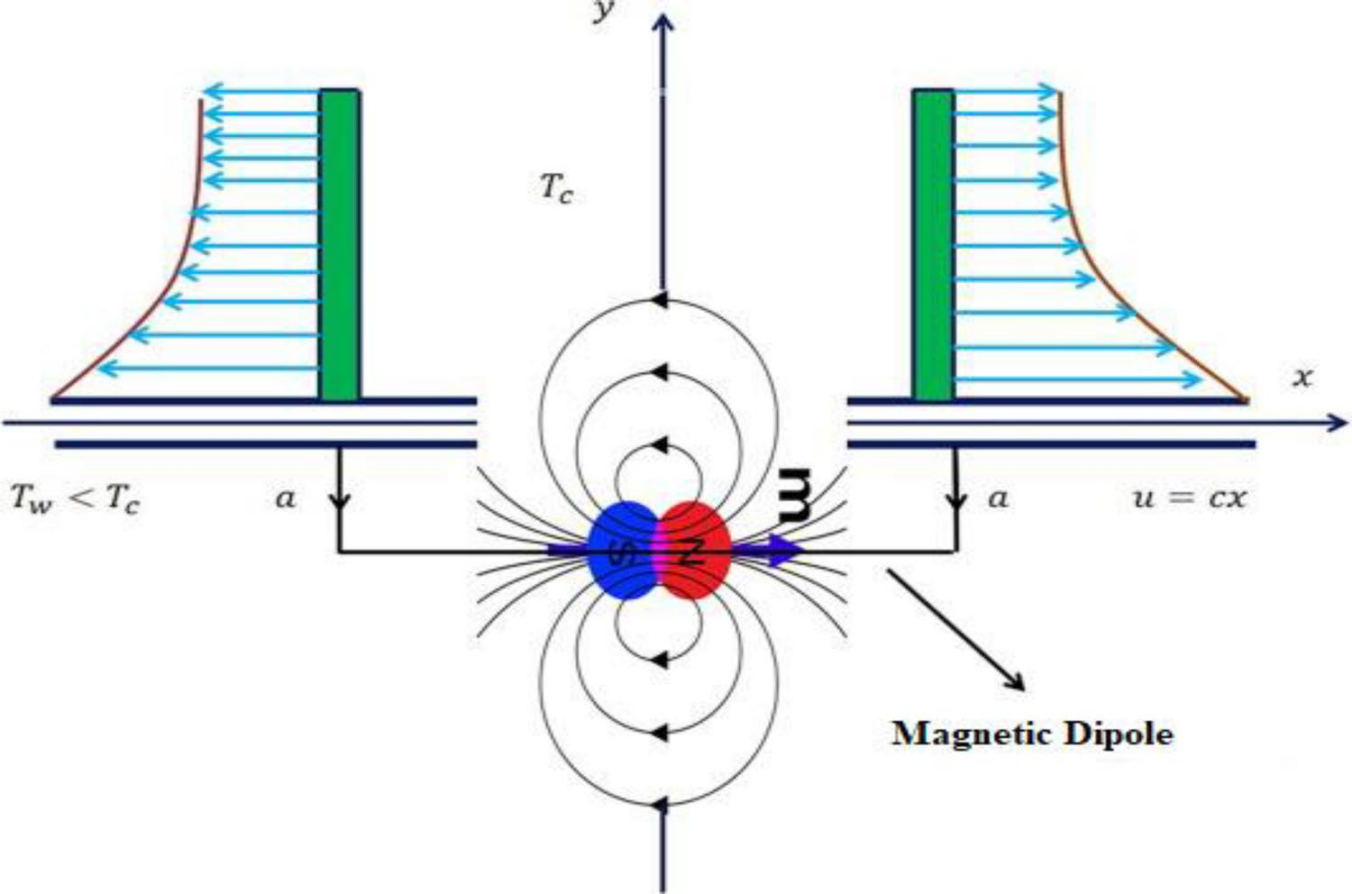
Schematic flow illustration.

We assume a 2D Oldroyd-B nanofluid flow model under the impact of the magnetic dipole over an extended surface along the *x-*direction. The distance between the magnetic dipole and the surface is “*a”* centered at the *y-*axis (Fig. 1).

To saturate the ferrofluid the magnetic dipole gives rise to the magnetic field along the positive *x-*direction. The temperature $T_{w}$ is taken at the surface nevertheless $T_{c}$ is considered far away from the sheet. Thus, the elements will not be magnetized until they outset for cooling in the neighborhood of the thermal boundary layer adjoining the surface. The Buongiorno nanofluid model is adopted here to signify the impacts of Brownian motion and thermophoretic effects. The amalgamation of activation energy with heat generation/absorption is also studied. At the surface, the melting heat boundary condition is adopted. The subsequent equations arise after applying the boundary layer theory because of the aforementioned assumptions ^34,45^:

1 $$ \frac{\partial u}{{\partial x}} + \frac{\partial v}{{\partial y}} = 0, $$

2 $$ \begin{aligned} & u\frac{\partial u}{{\partial x}} + v\frac{\partial u}{{\partial y}} + \lambda_{1} \left( {u^{2} \frac{{\partial^{2} u}}{{\partial x^{2} }} + v^{2} \frac{{\partial^{2} u}}{{\partial y^{2} }} + 2uv\frac{{\partial^{2} u}}{\partial x\partial y}} \right) = \upsilon_{f} \frac{{\partial^{2} u}}{{\partial y^{2} }} \\ & \quad + \,\nu \lambda_{2} \left( {u\frac{{\partial^{3} u}}{{\partial x\partial y^{2} }} + v\frac{{\partial^{3} u}}{{\partial y^{3} }} - \frac{\partial u}{{\partial x}}\frac{{\partial^{2} u}}{{\partial y^{2} }} - \frac{\partial u}{{\partial y}}\frac{{\partial^{2} v}}{{\partial y^{2} }}} \right) - \frac{{\lambda_{0} M}}{{\rho_{f} }}\frac{\partial H}{{\partial x}}, \\ \end{aligned} $$

3 $$ \begin{aligned} & u\frac{\partial T}{{\partial x}} + v\frac{\partial T}{{\partial y}} + \lambda_{3} \left\{ \begin{gathered} u\frac{\partial u}{{\partial x}}\frac{\partial T}{{\partial x}} + v\frac{\partial v}{{\partial y}}\frac{\partial T}{{\partial y}} + u^{2} \frac{{\partial^{2} T}}{{\partial x^{2} }} + v^{2} \frac{{\partial^{2} T}}{{\partial y^{2} }} \hfill \\ + 2uv\frac{{\partial^{2} T}}{\partial x\partial y} + u\frac{\partial v}{{\partial x}}\frac{\partial T}{{\partial y}} + v\frac{\partial u}{{\partial y}}\frac{\partial T}{{\partial x}} \hfill \\ \end{gathered} \right\} = \frac{{k_{f} }}{{\left( {\rho c_{p} } \right)_{f} }}\frac{{\partial^{2} T}}{{\partial y^{2} }} + \frac{{Q_{0} }}{{\left( {\rho c_{p} } \right)_{f} }}(T - T_{c} ) \\ & \quad + \tau \left[ {D_{B} \frac{\partial C}{{\partial y}}\frac{\partial T}{{\partial y}} + \frac{{D_{T} }}{{T_{c} }}\left( {\frac{\partial T}{{\partial y}}} \right)^{2} } \right] - \frac{{\lambda_{0} }}{{\left( {\rho c_{p} } \right)_{f} }}T\frac{\partial M}{{\partial T}}\left( {u\frac{\partial H}{{\partial x}} + v\frac{\partial H}{{\partial x}}} \right), \\ \end{aligned} $$

4 $$ u\frac{\partial C}{{\partial x}} + v\frac{\partial C}{{\partial y}} = D_{B} \frac{{\partial^{2} C}}{{\partial y^{2} }} + \frac{{\partial^{2} T}}{{\partial y^{2} }}\frac{{D_{T} }}{{T_{\infty } }} - k_{r}^{2} \left( {\frac{T}{{T_{\infty } }}} \right)^{n} \exp \left( {\frac{{ - E_{a} }}{kT}} \right)(C - C_{c} ). $$

The appropriate boundary conditions are specified by:

5 $$ \begin{aligned} & \left. u \right|_{y = 0} = u_{w} = cx,\, \, \left. v \right|_{y = 0} = 0, \, \left. T \right|_{y = 0} = T_{w} ,\left. C \right|_{y = 0} = C_{w} , \\ & k\left. {\frac{\partial T}{{\partial y}}} \right|_{y = 0} = \rho_{f} \left[ {\lambda^{*} + c_{s} (T_{w} - T_{0} )} \right]v(x,0), \\ & \left. u \right|_{y \to \infty } \to 0, \, \left. T \right|_{y \to \infty } \to T_{c} ,\, \, \left. v \right|_{y \to \infty } \to 0, \, \left. C \right|_{y \to \infty } \to C_{c} , \\ \end{aligned} $$

## Magnetic dipole appearance

The magnetic dipole scalar potential $\Phi$ is presented by ^45,46^:

6 $$ \Phi = \frac{\gamma x}{{2\pi (x^{2} + (y + a)^{2} )}}, $$

Here, $\gamma$ signifies the strength of the magnetic dipole. The magnetic force is characterized by its scalar function considering the conservation of the magnetic force field. The elements of the magnetic field $H$ are given by:

7 $$ \begin{aligned} & \frac{\partial H}{{\partial x}} = - \frac{\partial \Phi }{{\partial x}} = \frac{{\gamma \left[ {x^{2} - (y + a)^{2} } \right]}}{{2\pi (x^{2} + (y + a)^{2} )^{2} }}, \\ & \frac{\partial H}{{\partial y}} = - \frac{\partial \Phi }{{\partial y}} = \frac{2\gamma x(y + a)}{{2\pi (x^{2} + (y + a)^{2} )^{2} }}. \\ \end{aligned} $$

8 $$ H = \sqrt {\left( {\frac{\partial \Phi }{{\partial x}}} \right)^{2} + \left( {\frac{\partial \Phi }{{\partial y}}} \right)^{2} } , $$

Equation (8) gives

9 $$ \begin{aligned} & \frac{\partial H}{{\partial x}} = \frac{ - 2x\gamma }{{2\pi (y + a)^{4} }}, \\ & \frac{\partial H}{{\partial y}} = \frac{\gamma }{2\pi }\left( {\frac{ - 2}{{(y + a)^{3} }} + \frac{{4x^{2} }}{{(y + a)^{5} }}} \right). \\ \end{aligned} $$

The magnetic effect in terms of temperature is given by:

10 $$ M = K(T_{c} - T), $$

where $K$ characterize the gyromagnetic coefficient. For the ferromagnetic phenomenon, the applied magnetic field must be nonhomogeneous and $T_{c} > T.$ Also, it is pertinent to say that when the ferrofluid attains a temperature of $T_{c}$, no further magnetization is required. However, as depicted from Eq. (10), the fluid can’t attain the temperature $T_{c}$ away from the surface.

## Similarity transformation

Applying the subsequent dimensionless transformation to convert the above-mentioned system takes the form:

11 $$ \begin{aligned} & \psi (\xi ,{\rm O} \\ \\ \end{aligned} $$

To have a solution independent of variables, it is required to have all defined parameters dimensionless. Using the aforementioned transformations, the Eckert number will have the subsequent form:

12 $$ E_{c} = \frac{{u_{w}^{2} }}{{C_{p} (T_{w} - T_{c} )}} = \frac{{c^{2} x^{2} }}{{C_{p} (T_{w} - T_{c} )}} = E_{c} (x). $$

For $E_{c}$ to be independent of $x$, the temperature $T_{w}$ is defined in the following form ^59^:

13 $$ T_{w} = T_{c} + T_{0} x^{2} , $$

where $T_{0}$ is a constant. Else, the solutions obtained are only locally similar. The requirement of Eq. (1) is fulfilled trivially, and Eqs. (2) to (4) take the form

14 $$ f^{\prime \prime \prime } + \beta_{2} (f^{\prime \prime 2} - ff^{iv} ) + ff^{\prime\prime} - f^{\prime 2} - \beta_{1} (f^{2} f^{\prime \prime \prime } - 2ff^{\prime}f^{\prime\prime}) - \frac{2\beta }{{(\eta + \alpha )^{4} }}\theta = 0, $$

15 $$ \theta_{1} ^{\prime\prime} + \Pr \left\{ \begin{gathered} \left( \begin{gathered} f\theta_{1} ^{\prime} + \phi^{\prime}\theta_{1} ^{\prime}N_{b} \hfill \\ N_{t} \theta_{1} ^{{\prime}{2}} + D_{c} \theta_{1} \hfill \\ \end{gathered} \right) - \gamma^{*} \left( {f^{2} \theta_{1} ^{\prime\prime} + ff^{\prime}\theta_{1} ^{\prime}} \right) + \hfill \\ \, \hfill \\ \end{gathered} \right\} + \frac{{2\lambda \beta (\theta_{1} - \varepsilon )f}}{{(\eta + \alpha )^{3} }} = 0, $$

16 $$ \theta^{\prime\prime}_{2} + \Pr \left\{ \begin{gathered} \left( \begin{gathered} f\theta^{\prime}_{2} + \phi^{\prime}\theta^{\prime}_{2} N_{b} \hfill \\ N_{t} \theta_{2} ^{{\prime}{2}} + D_{c} \theta_{2} \hfill \\ \end{gathered} \right) - \gamma^{*} \left( {f^{2} \theta^{\prime\prime}_{2} + ff^{\prime}\theta^{\prime}_{2} } \right) + \hfill \\ \, \hfill \\ \end{gathered} \right\} + \frac{{2\lambda \beta \theta_{2} f}}{{(\eta + \alpha )^{3} }} - \lambda (\theta_{1} - \varepsilon )\beta \left[ {\frac{{2f^{\prime}}}{{(\eta + \alpha )^{4} }} + \frac{4f}{{(\eta + \alpha )^{5} }}} \right] = 0, $$

17 $$ \phi^{\prime\prime} + \frac{{N_{t} }}{{N_{b} }}\theta^{\prime\prime} + S_{c} f\phi^{\prime} - R_{c} \phi (1 + \alpha_{1} \theta )^{m} \exp \left( {\frac{ - E}{{1 + \alpha_{1} \theta }}} \right) = 0, $$

and the boundary condition (5) yields the following form

18 $$ \begin{aligned} & f^{\prime}(0) = 1,\,\Pr f(0) + M_{a} \theta^{\prime}_{1} (0) = 0,\,\theta_{1} (0) = 1,\,\theta_{2} (0) = 0;g(0) = 1, \\ & \, f^{\prime}(\eta ) = 0,\,f^{\prime\prime}(\eta ) = 0, \, \theta_{1} (\eta ) = 0, \, \theta_{2} (\eta ) = 0,g(\eta ) = 0,\quad {\text{as}}\,\,\eta \to \infty , \\ \end{aligned} $$

where the parameters mentioned above are translated as:

19 $$ \begin{aligned} & M_{a} = \frac{{c_{f} (T_{c} - T_{w} )}}{{\lambda^{*} + c_{s} (T_{w} - T_{0} )}},{\text{ Pr}} = \frac{{\nu_{f} }}{{\alpha_{f} }},\varepsilon = \frac{{T_{c} }}{{T_{c} - T_{w} }},\beta = \frac{{\gamma \rho_{f} \lambda_{0} K}}{{2\pi \mu_{0}^{2} }}(T_{c} - T_{w} ), \\ & \alpha = a\sqrt {\frac{{c\rho_{f} }}{{\mu_{0} }}} , \, D_{c} = \frac{{Q_{0} }}{{c(\rho C_{p} )_{f} }}, \, S_{c} = \frac{{\nu_{f} }}{{D_{B} }},\gamma^{*} = \lambda_{3} c,E = \frac{{ - E_{a} }}{{kT_{c} }},Ec = \frac{{c^{2} }}{{C_{p} T_{0} }}, \\ & N_{t} = \frac{{\tau D_{T} (T_{w} - T_{c} )}}{{cT_{c} }}, \, N_{b} = \frac{{\tau D_{B} (C_{w} - C_{c} )}}{{\upsilon_{f} }},R_{c} = \frac{{k_{r}^{2} }}{c},\beta_{1} = \lambda_{1} c,\,\,\,\beta_{2} = \lambda_{2} c, \\ \end{aligned} $$

## Local Nusselt and Sherwood numbers

The dimensional form of the $Nu_{x}$ (the Nusselt number) and $Sh_{x}$ (the Sherwood number)are characterized as:

20 $$ Nu_{x} = \left. {\frac{{xq_{m} }}{{ - k(T_{c} - T_{w} )}}} \right|_{y = 0} ,\quad Sh_{x} = \left. {\frac{{xq_{n} }}{{ - D_{B} (C_{c} - C_{w} )}}} \right|_{y = 0} , $$

with

21 $$ q_{m} = \left. { - k\frac{\partial T}{{\partial y}}} \right|_{y = 0} ,q_{n} = \left. { - k\frac{\partial C}{{\partial y}}} \right|_{y = 0} $$

Dimensionless forms both the Nusselt number and the Sherwood number are:

22 $$ Nu_{x} Re_{x}^{ - 1/2} = \left[ {\theta^{\prime}_{1} (0) + \xi^{2} \theta^{\prime}_{2} (0)} \right],ShRe_{x}^{ - 1/2} = - g^{\prime}(0). $$

where ${\text{Re}}_{x} = \frac{{cx^{2} }}{{\nu_{f} }}\,\,\,$ is the local Reynolds number.

## Entropy generation

The volumetric rate of local entropy generation over the stretching sheet is given by ^60^:

23 $$ \begin{aligned} S_{gen}^{\prime \prime \prime } & = \underbrace {{\frac{{k_{f} }}{{T_{c}^{2} }}\left( {\frac{\partial T}{{\partial y}}} \right)^{2} }}_{heat\,\,transfer\,\,irreversibility} + \underbrace {{\frac{\mu }{{T_{c} }}\left[ {\left( {\frac{\partial u}{{\partial y}}} \right)^{2} + \lambda_{2} \left( {u\frac{\partial u}{{\partial y}}\frac{{\partial^{2} u}}{\partial x\partial y} + v\frac{{\partial^{3} u}}{{\partial y^{3} }}} \right)} \right]}}_{fluid\,\,friction\,\,irreversibility} \\ & \quad + \underbrace {{\frac{RD}{{T_{c} }}\left( {\frac{\partial C}{{\partial y}}} \right)\left( {\frac{\partial T}{{\partial y}}} \right) + \frac{RD}{{C_{c} }}\left( {\frac{\partial C}{{\partial y}}} \right)^{2} }}_{Diffusive\,\,irreversibility}, \\ \end{aligned} $$

Utilizing the similarity transformation (11), the entropy generation takes the form:

24 $$ \begin{aligned} N_{G} (\eta ) & = \frac{{S_{gen}^{\prime \prime \prime \prime } }}{{S_{0}^{\prime \prime \prime \prime } }} = \frac{{S_{gen}^{\prime \prime \prime \prime } }}{{k\left( {\Delta T} \right)^{2} /L^{2} T_{c}^{2} }} = \alpha_{1} \theta^{\prime 2} + {\text{Re}}_{x} Br\left[ {f^{\prime \prime 2} + \beta_{2} \left( {f^{\prime}f^{\prime \prime 2} - ff^{\prime\prime}f^{\prime \prime \prime } } \right)} \right] \\ & \quad + \,L\frac{{\alpha_{2} }}{{\alpha_{1} }}g^{\prime 2} + L\theta^{\prime}g^{\prime}, \\ \end{aligned} $$

where

25 $$ \alpha_{1} = \frac{\Delta T}{{T_{c} }},Br = \frac{{\mu_{f} c^{2} }}{{C_{p} k_{f} T_{0} }},\alpha_{2} = \frac{\Delta C}{{C_{c} }},L = \frac{RD\Delta C}{{k_{f} }}. $$

## Numerical solution

The numerical solution of Eqs. (14) to (17) corresponding to boundary conditions (18) is accomplished via the bvp4c MATLAB function. To achieve this objective, the subject system of higher-order differential equations is transformed into the system of order one. The tolerance of the presented problem is taken $10^{ - 5}$. The appropriate finite estimate of $\eta \to \infty$ as $\eta_{\infty } = \eta = 7$ is taken considering arising parameter values.

26 $$ \begin{aligned} & y_{1} = f,\,\,y_{2} = f^{\prime},\,\,y_{3} = f^{\prime\prime},y_{4} = f^{\prime \prime \prime } ,\,\,\,\,\,\theta_{1} = y_{5} ,\theta_{1} ^{\prime} = y_{6} ,\, \\ & \theta_{2} = y_{6} ,\theta_{7} ^{\prime} = y_{8} ,\,\,\,\,\,\,\,g = y_{9} ,\,\,\,g^{\prime} = y_{10} . \\ \end{aligned} $$

Using the above expressions in MATLAB bvp4c we have the following set of first-order equations:

27 $$ yy1 = \frac{1}{{y_{1} }}\left( { - y_{4} - \beta_{2} y_{3}^{2} - y_{1} y_{3} + y_{2}^{2} + \beta_{1} \left( {y_{1}^{2} y_{4} - 2y_{1} y_{2} y_{3} + \frac{2\beta }{{(\eta + \alpha )^{4} }}y_{5} } \right)} \right); $$

28 $$ yy2 = \left( {\frac{1}{{(1 - \gamma y_{1}^{2} }}} \right)\left( { - \Pr \left( {\left( {y_{1} y_{6} + y_{9} y_{6} N_{b} + N_{t} y_{6}^{2} + D_{c} y_{5} } \right) - \gamma \left( {y_{1} y_{2} y_{6} } \right)} \right) - \frac{{2\lambda \beta \left( {y_{5} - \varepsilon } \right)y_{5} }}{{(\eta + \alpha )^{3} }}} \right); $$

29 $$ yy3 = \left( {\frac{1}{{(1 - \gamma y_{2}^{2} }}} \right)\left( \begin{gathered} - \Pr \left( {\left( {y_{1} y_{8} + y_{10} y_{8} N_{b} + N_{t} y_{8}^{2} + D_{c} y_{7} } \right) - \gamma \left( {y_{1} y_{2} y_{8} } \right)} \right) - \frac{{2\lambda \beta y_{7} y_{1} }}{{(\eta + \alpha )^{3} }} \hfill \\ + \lambda \left( {y_{5} - \varepsilon } \right)\beta \left( {\frac{{2y_{2} }}{{(\eta + \alpha )^{4} }} + 4\frac{{y_{1} }}{{(\eta + \alpha )^{5} }}} \right) \hfill \\ \end{gathered} \right); $$

30 $$ yy4 = \frac{{ - N_{t} }}{{N_{b} }}yy2 - Scy_{1} y_{9} + R_{c} y_{9} (\eta + \alpha )^{m} \exp \left( {\frac{ - E}{{(\eta + \alpha_{1} y_{5} )}}} \right); $$

with the transformed BCs

31 $$ \begin{aligned} & y_{0} (1) - 1;\Pr y_{0} (1) + M_{a} y_{0} (5);\,\,\,y_{0} (5) - 1;\,\,y_{0} (7)\,\,;y_{0} (9) - 1; \\ & y_{\inf } (2);\,y_{\inf } (5);y_{\inf } (7);\,y_{\inf } (9); \\ \end{aligned} $$

A grid independence test is performed for the Sherwood number. It can be visualized from the Table 1 that the grid size 300*300 is suitable for the system to be grid independent. For this fixed value, the system seems to be grid free.

**Table 1:** Grid free analysis for the Sherwood number.

Serial No	Grid size	$Sh_{x}$
1	10 × 10	2.4042
2	50 × 50	2.4057
3	70 × 70	2.4058
4	90 × 90	2.4062
5	100 × 100	2.4063
6	200 × 200	2.4064
7	300 × 300	2.4064

## Results and discussion

The primary aim of this segment is to deliberate the arising parameters’ influence on the associated distributions in mathematical modeling. The acceptable ranges of the involved parameters are $\left( {0.4 \le \beta \le 2.2} \right),\left( {0.1 \le \beta_{1} \le 1.3} \right),\left( {0.2 \le \lambda \le 0.8} \right),\left( {0.1 \le \gamma * \le 0.4} \right),\left( {0.1 \le Dc \le 0.4} \right),$ $\left( {0.1 \le N_{b} \le 0.3} \right),\left( {0.0 \le N_{t} \le 0.3} \right),\left( {0.1 \le R_{c} \le 0.4} \right),\left( {0.1 \le M_{a} \le 09} \right),\left( {1.0 \le \alpha_{1} \le 4.0} \right),$

**Figure 2:**
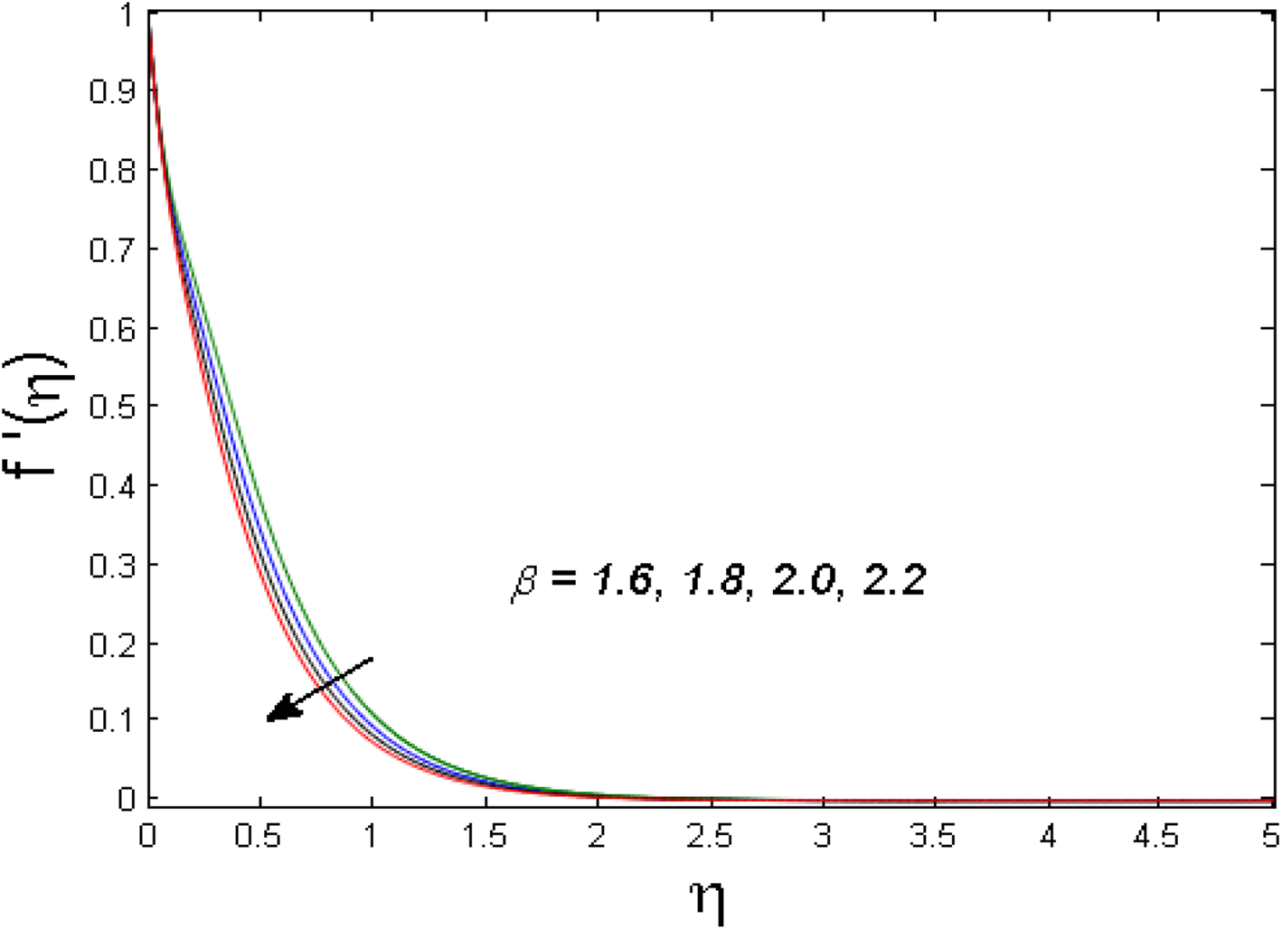
$f^{\prime}(\eta )$ versus varied estimates of $\beta$.

**Figure 3:**
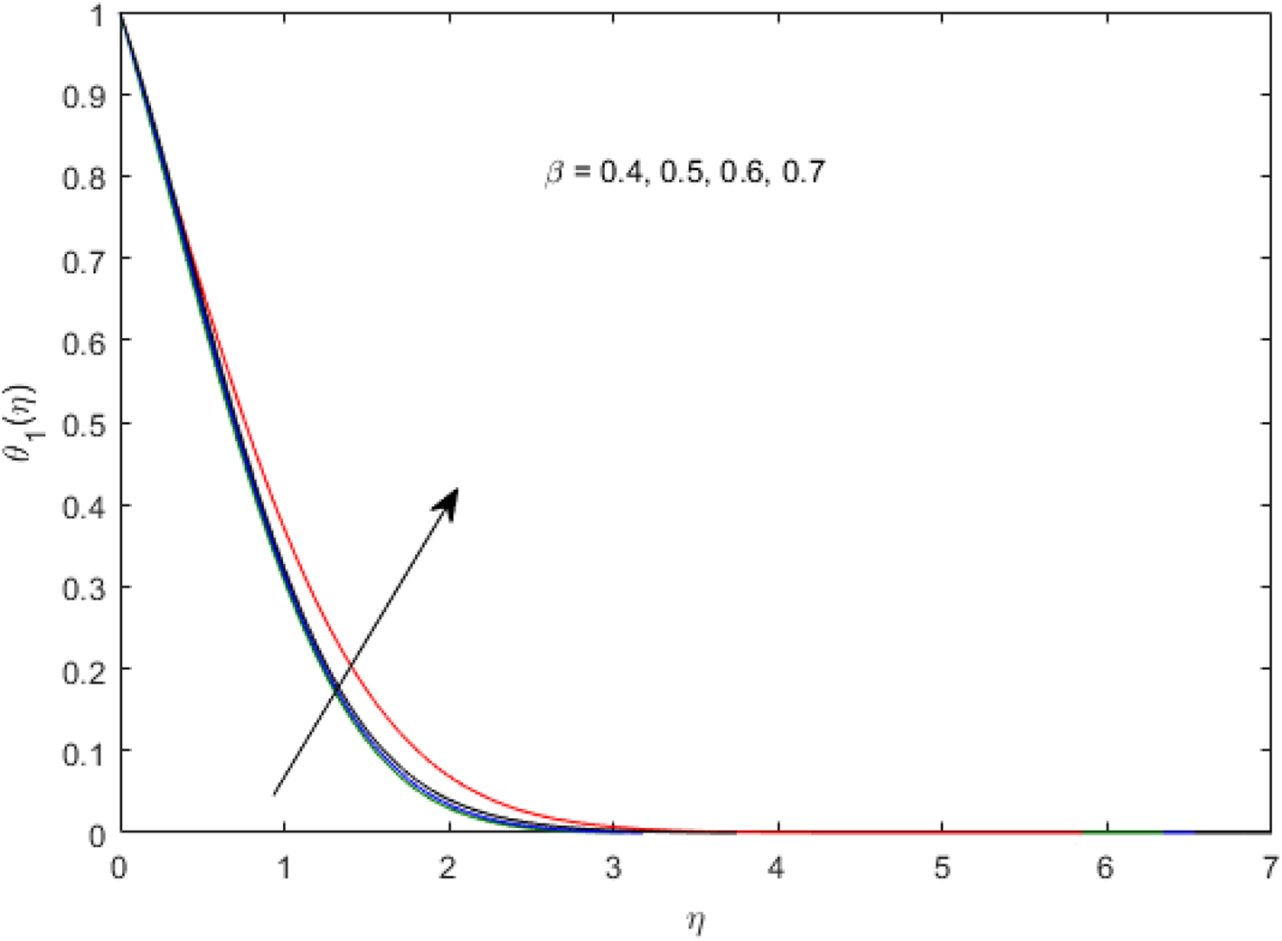
$\theta_{1} (\eta )$ versus varied estimates of $\beta$.

**Figure 4:**
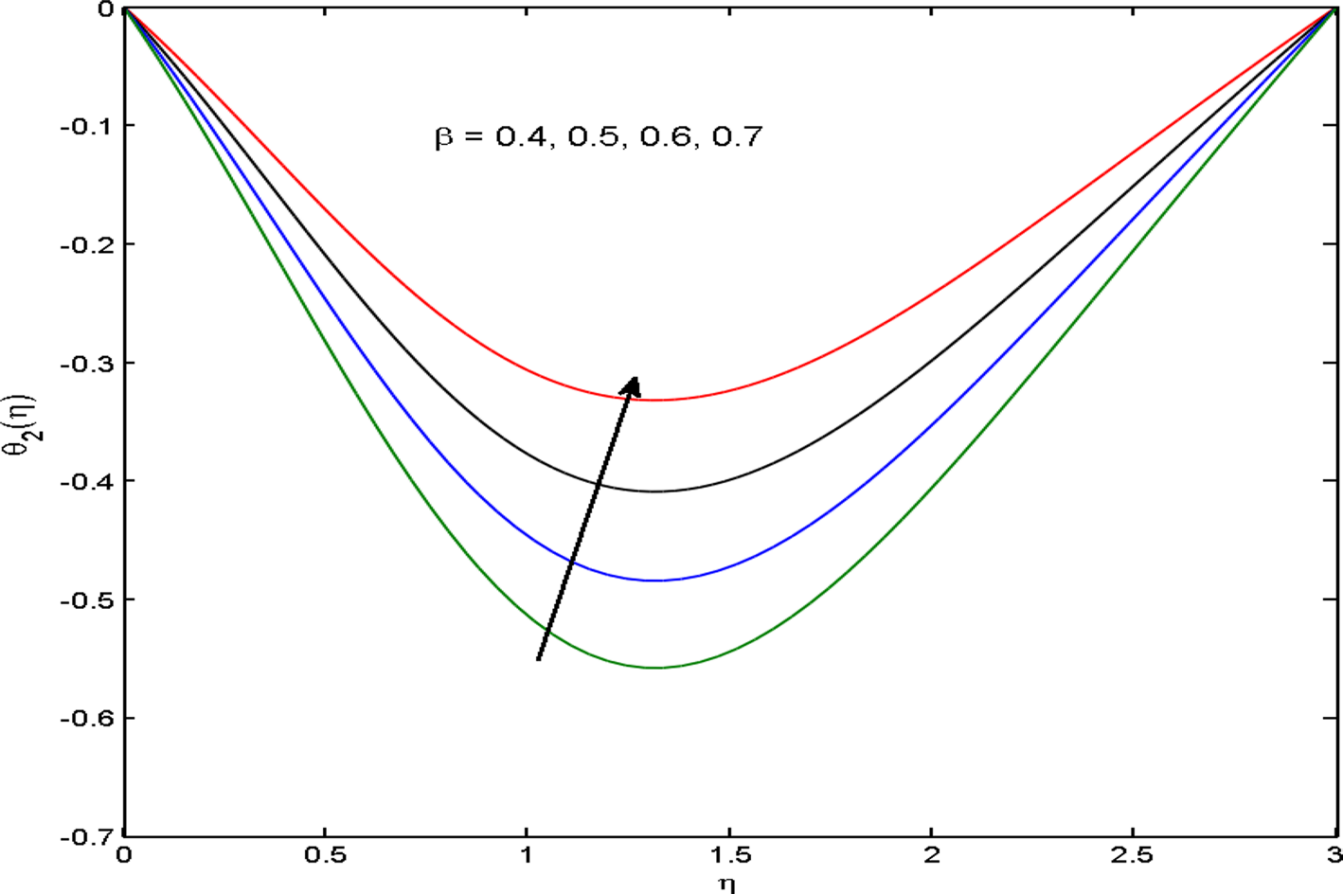
$\theta_{2} (\eta )$ versus varied estimates of $\beta$.

**Figure 5:**
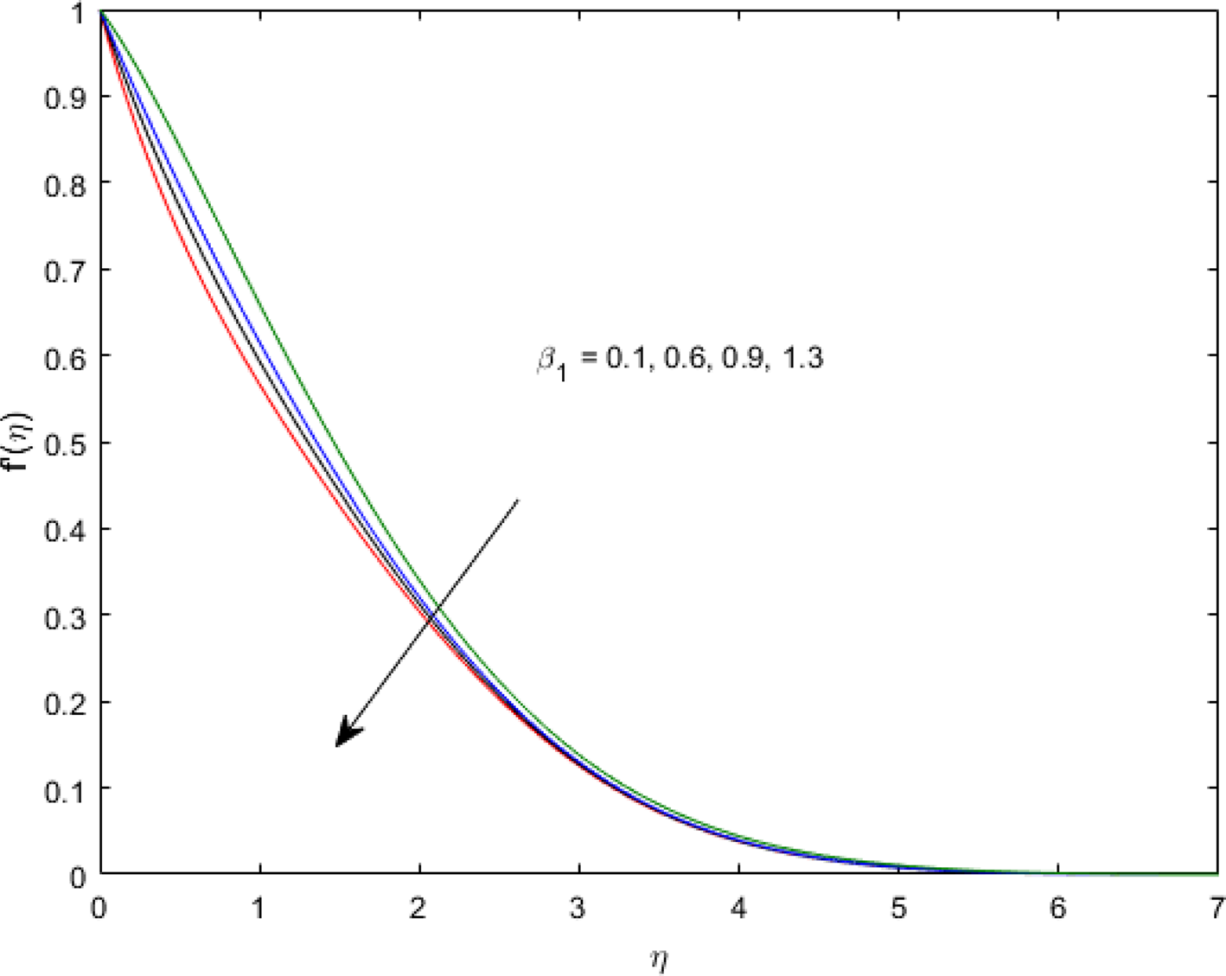
$f^{\prime}(\eta )$ versus varied estimates of $\beta_{1}$.

**Figure 6:**
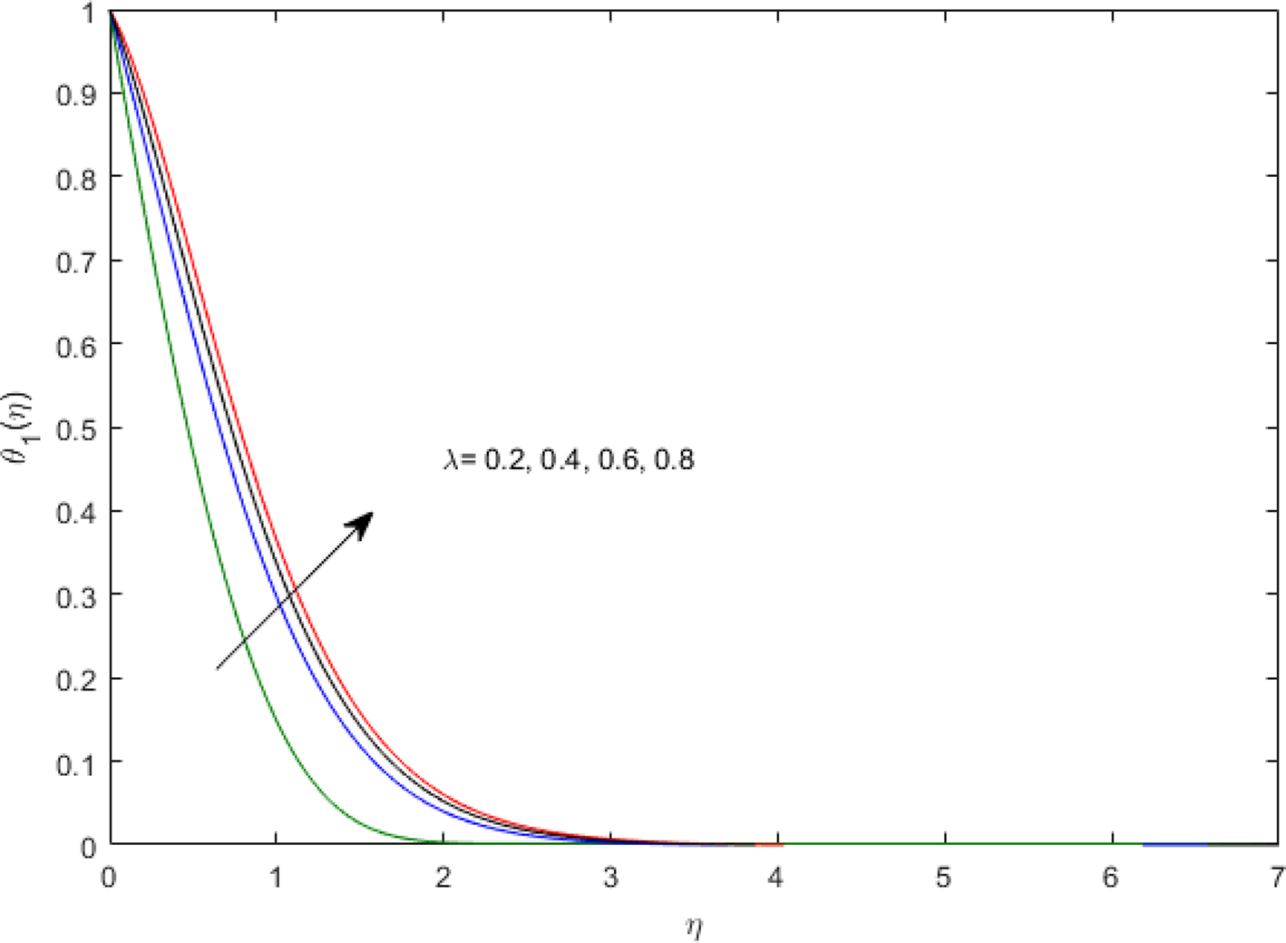
$\theta_{1} (\eta )$ versus varied estimates of $\lambda$.

**Figure 7:**
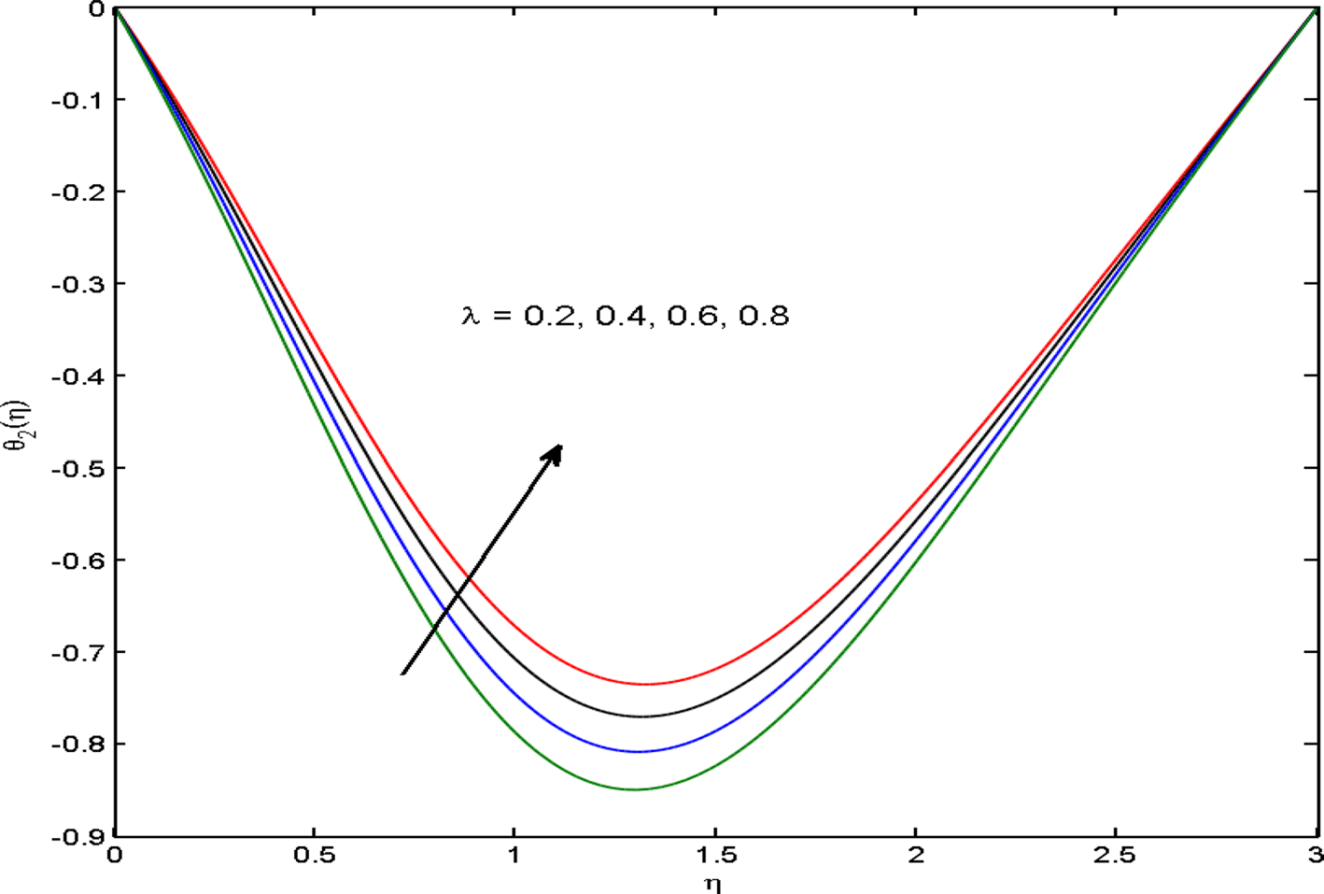
$\theta_{2} (\eta )$ versus varied estimates of $\lambda$.

**Figure 8:**
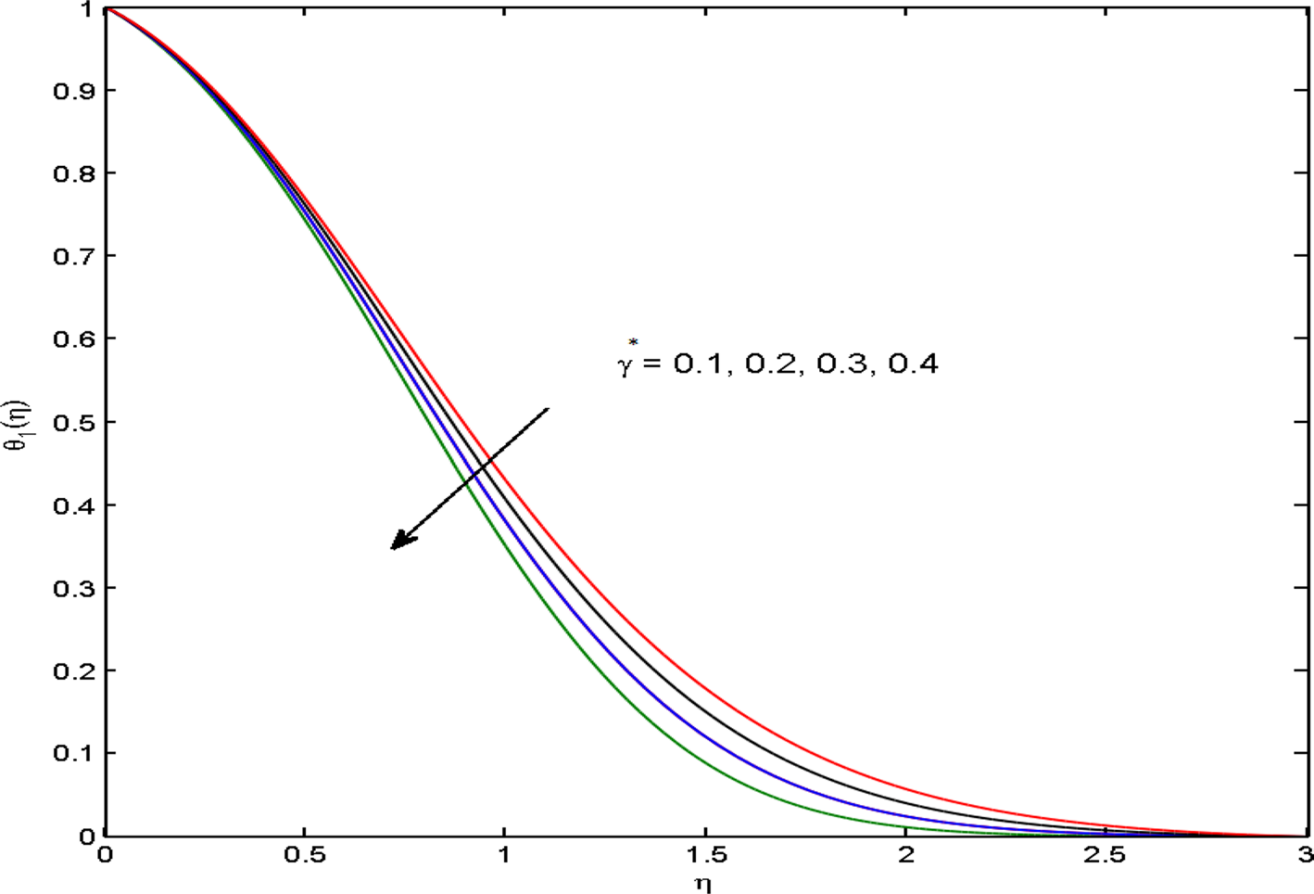
$\theta_{1} (\eta )$ versus varied estimates of $\gamma *$.

**Figure 9:**
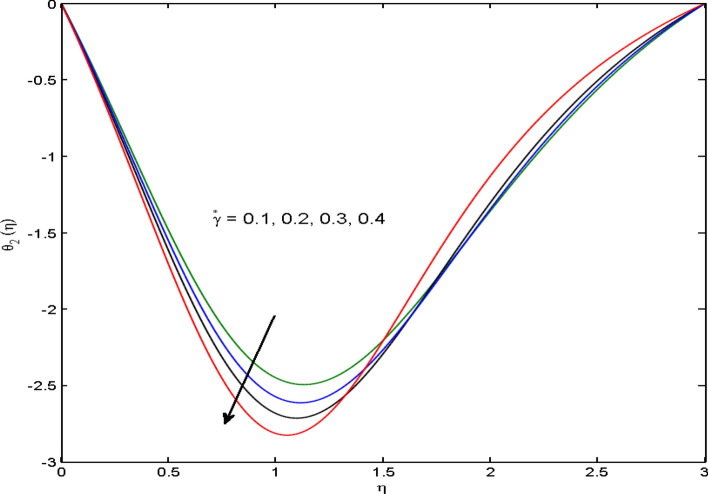
$\theta_{2} (\eta )$ versus varied estimates of $\gamma *$.

**Figure 10:**
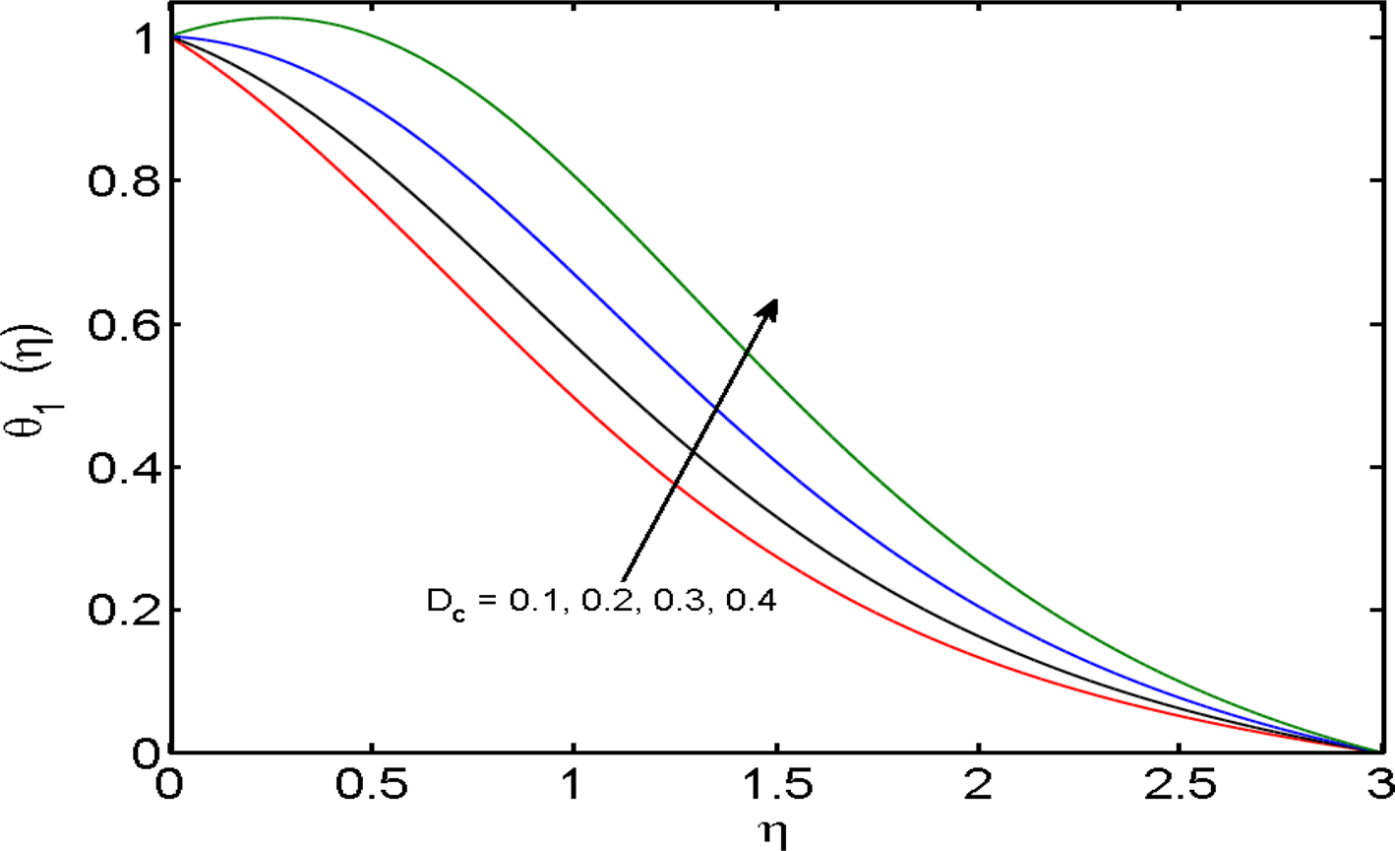
$\theta_{1} (\eta )$ versus varied estimates of $D_{c}$.

**Figure 11:**
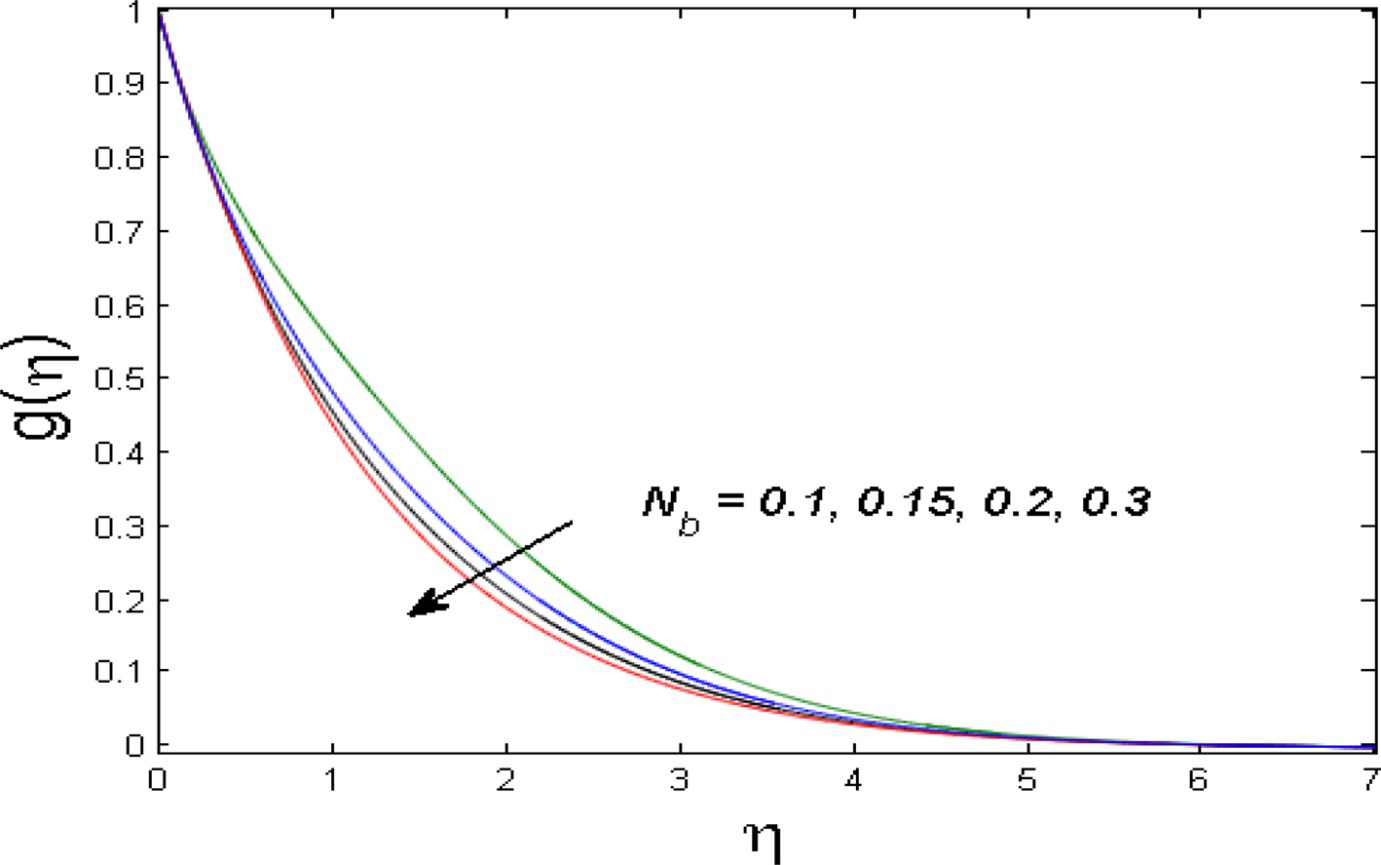
$g(\eta )$ versus varied estimates of $N_{b}$.

**Figure 12:**
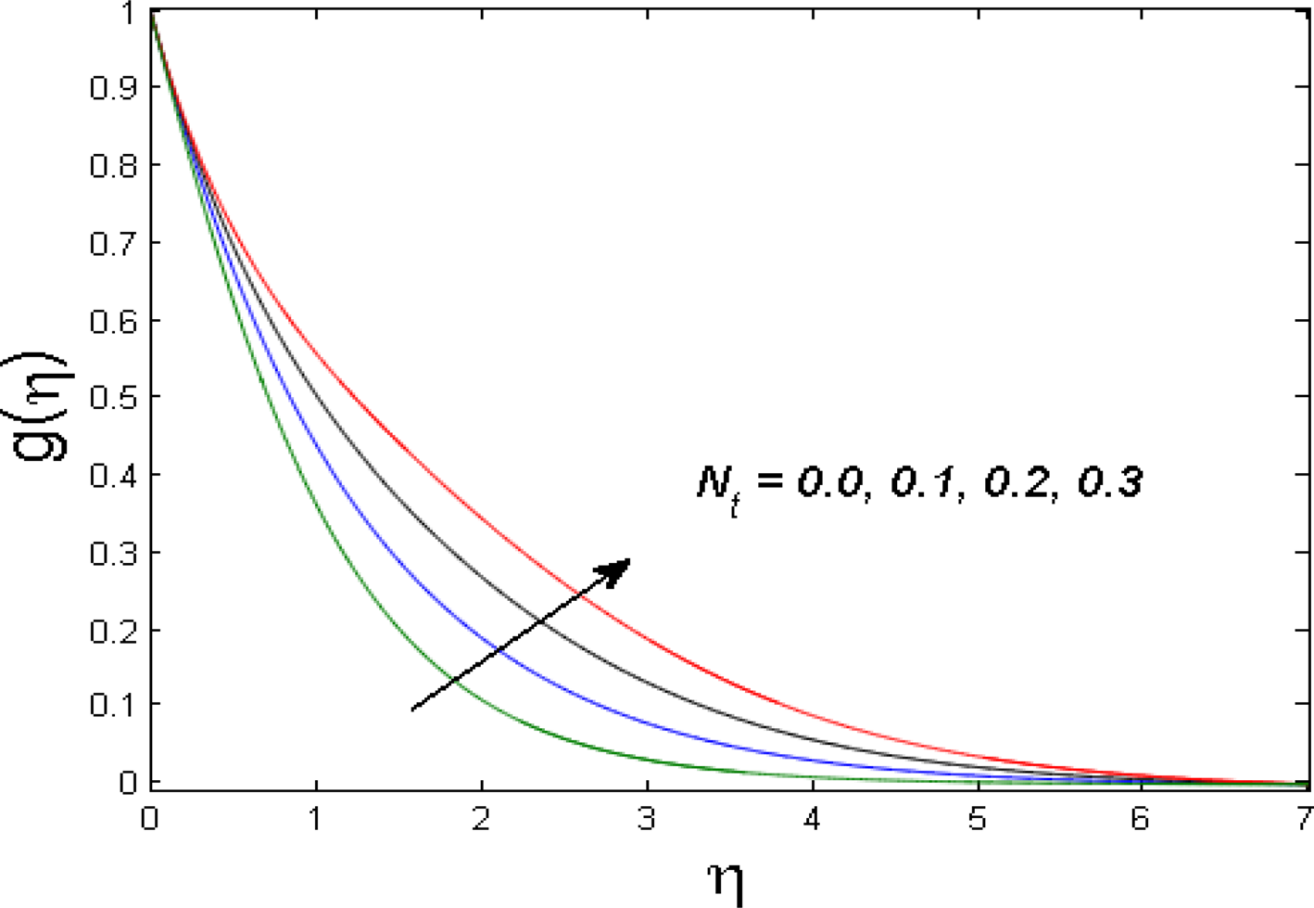
$g(\eta )$ versus varied estimates of $N_{t}$.

**Figure 13:**
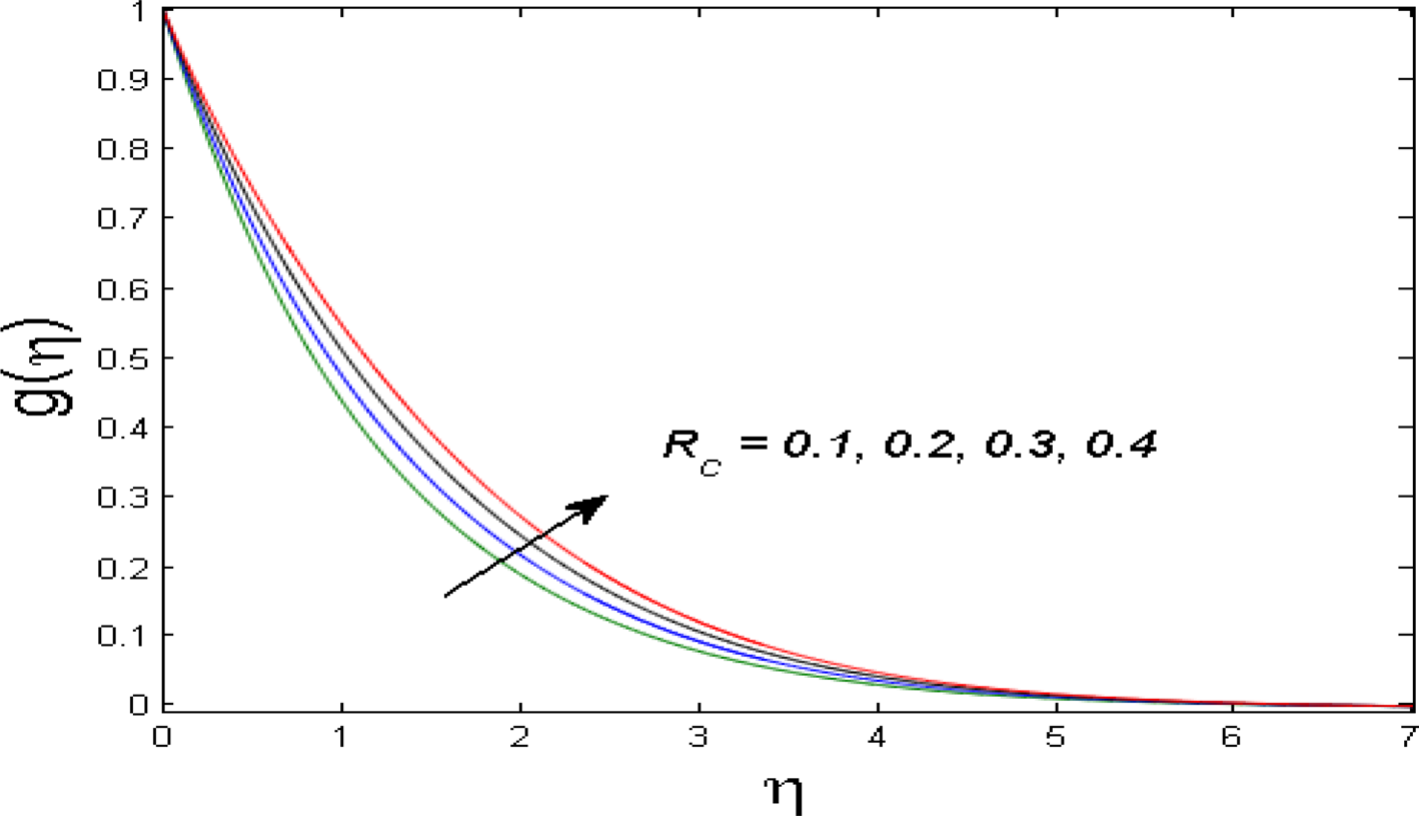
$g(\eta )$ versus varied estimates of $R_{c}$.

**Figure 14:**
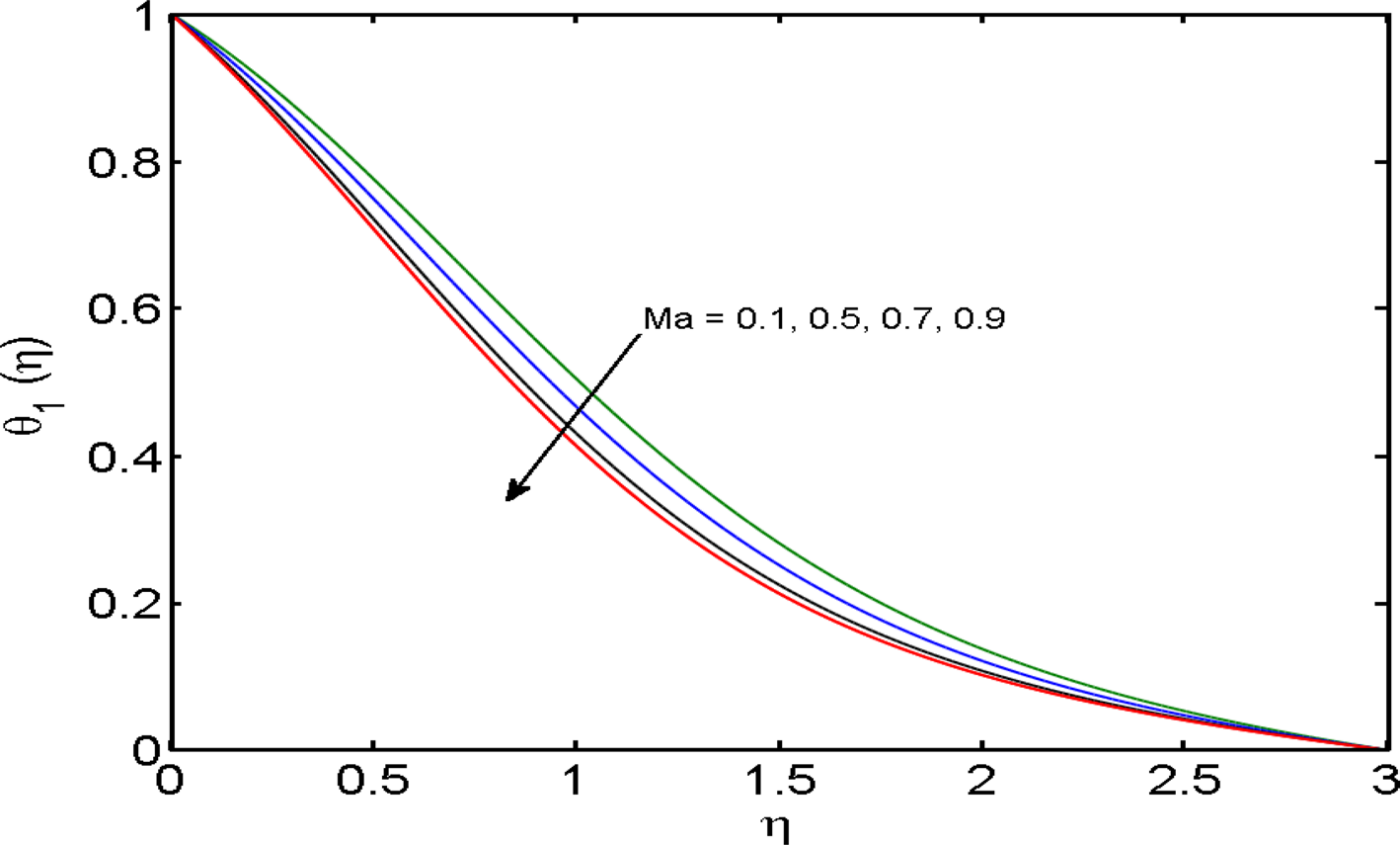
$\theta_{1} (\eta )$ versus varied estimates of $M_{a}$.

**Figure 15:**
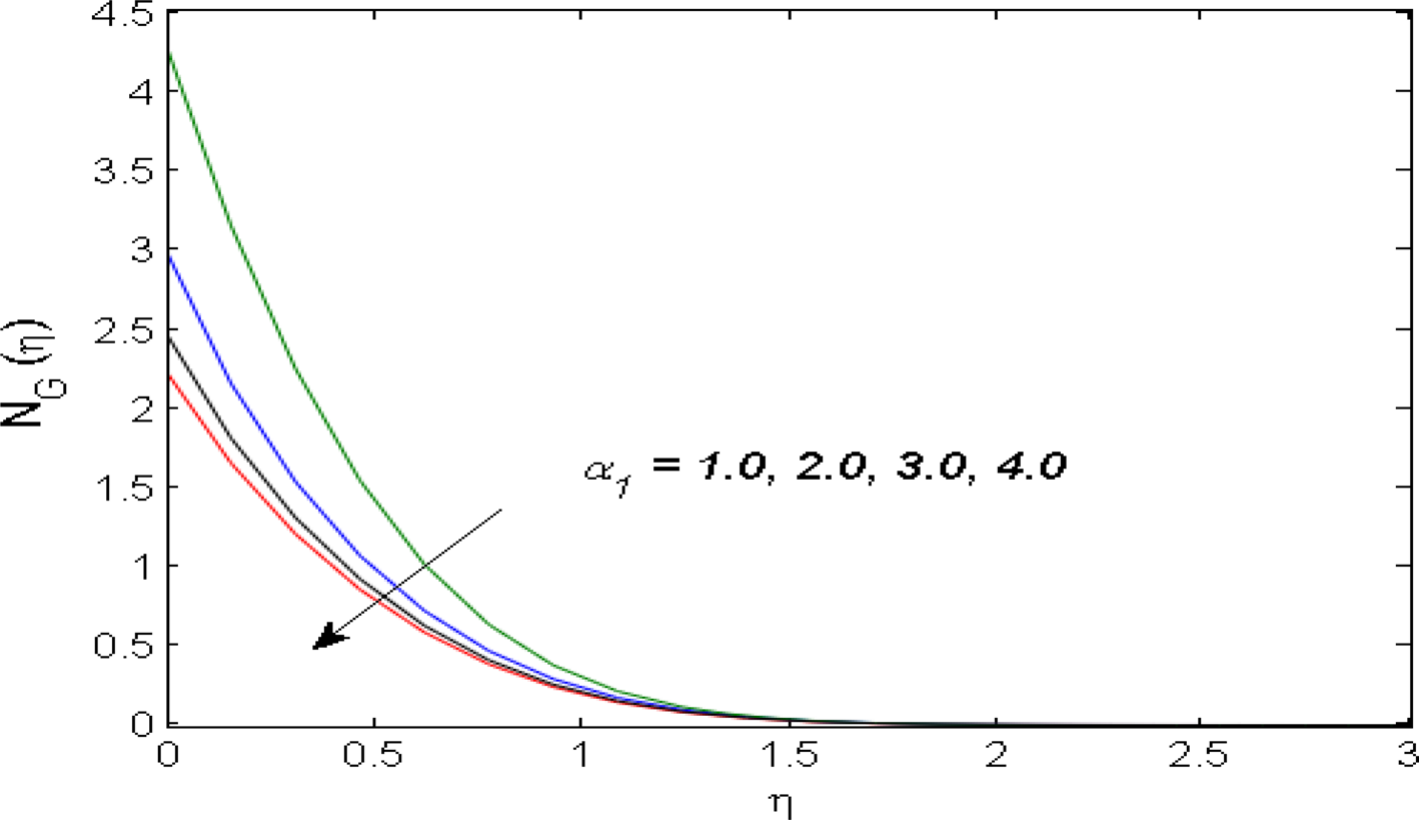
$N_{G} (\eta )$ versus varied estimates of $\alpha_{1}$.

**Figure 16:**
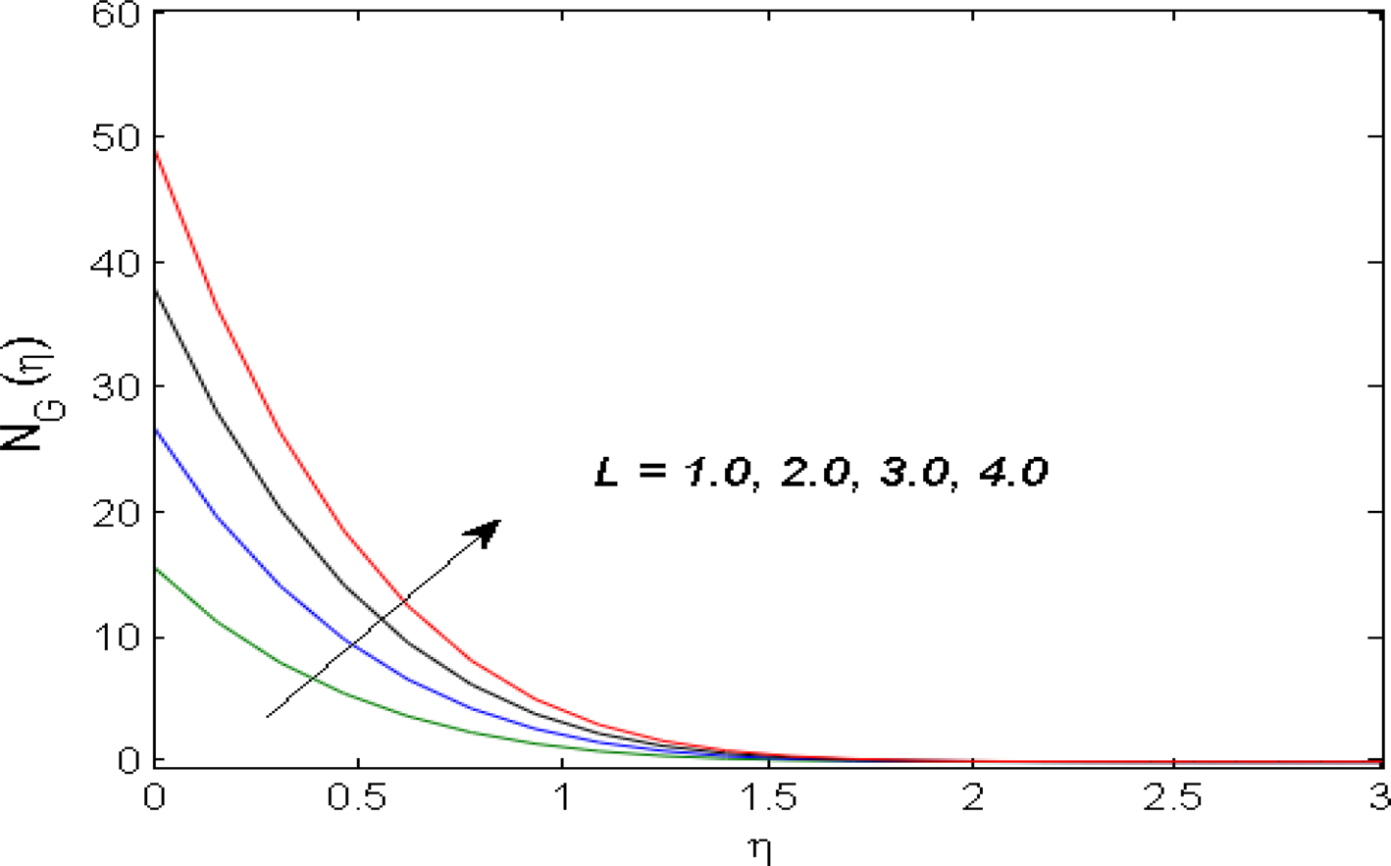
$N_{G} (\eta )$ versus varied estimates of $L$.

**Figure 17:**
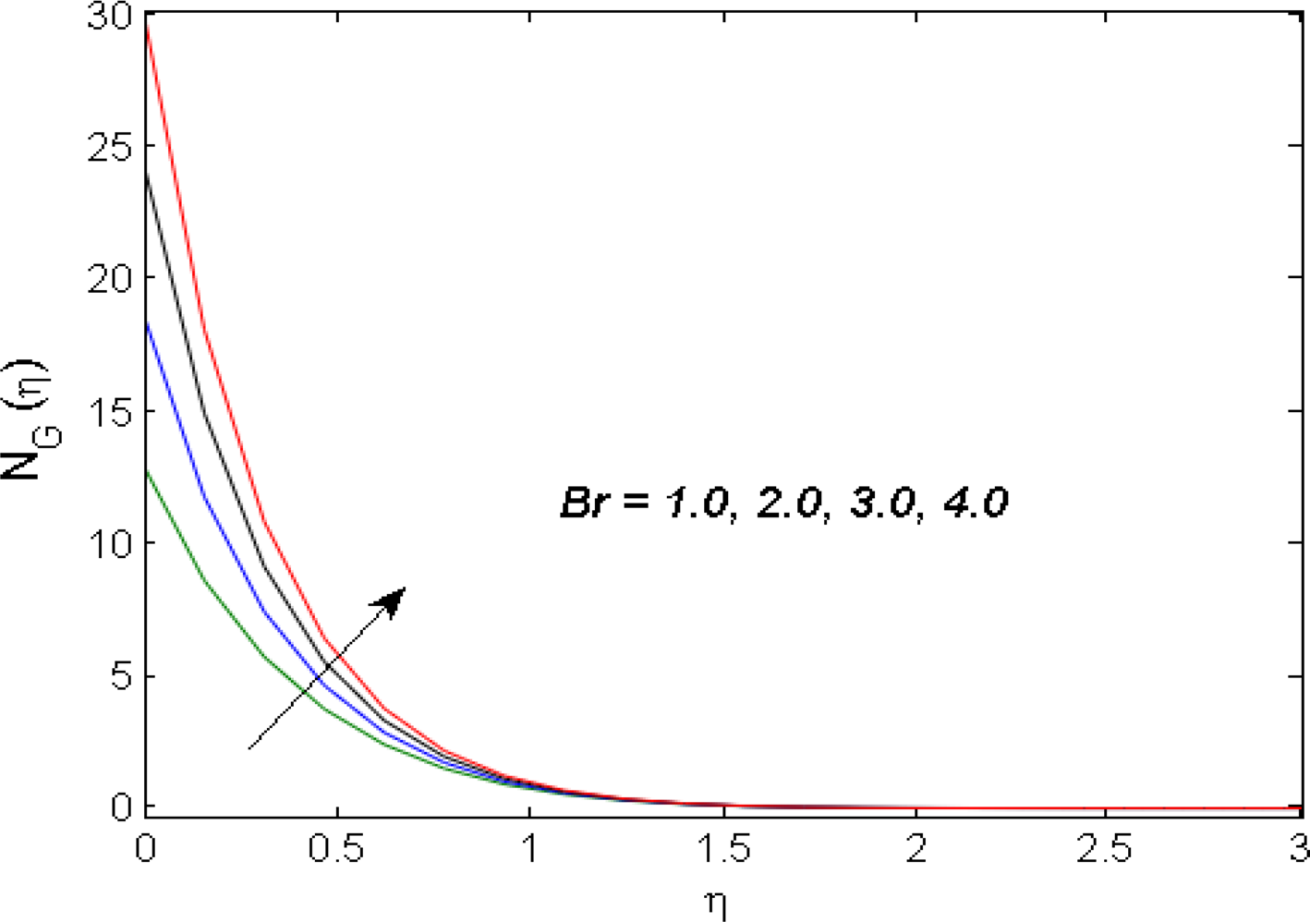
$N_{G} (\eta )$ for versus estimates of $B_{r}$.

$\left( {1.0 \le L \le 4.0} \right),\left( {1.0 \le B_{r} \le 4.0} \right),$ and these values are chosen on the basis that gives the best graphical resolution. Figures 2, 3 and 4 depict the velocity and temperature distributions’ behavior versus the ferrohydrodynamic interaction parameter $\beta$. The fluid will be more viscous with strong adhesive forces owing to ferrohydrodynamic interaction and a retardation in the velocity is perceived. Nevertheless, the temperature of the fluid on the contrary is aggravated for growing $\beta$. To witness the upshot of the material parameter $\beta_{1}$ on the velocity profile Fig. 5 is drawn. It is palpable from the figure that velocity and the boundary layer thickness are diminished are large estimates of $\beta_{1} .$ This logic for this decrease in the velocity is because of the increase in the non-dimensional relaxation time constant is that the large value of $\beta_{1}$ causes a slower recuperation rate. Figures 6 and 7 are sketched to show the relationship of viscous dissipation $\lambda$ and the temperature distributions. It is an acknowledged reality that the temperature of the fluid improves once the impact of viscous dissipation is introduced. This also abides by the definition of viscous dissipation. The thermal relaxation time parameter $\gamma^{*}$ influence on both temperature profiles is exhibited in Figs. 8 and 9. It is noticed that fluid temperature falls due to distended thermal relaxation time. This phenomenon requires more time to transport heat to the adjacent particles. That is why fluid temperature is dropped. Figure 10 highlights the heat generation parameter $D_{c}$ impact on the temperature of the fluid. Enhanced fluid temperature is noticed for $D_{c}$. The rate of heat transfer is triggered from the face of the surface to the liquid under consideration. Thus, the augmented temperature of the fluid is recognized. Figures 11 and 12 are drawn to underline how the concentration of the fluid is affected by the Brownian motion and thermophoretic parameters respectively. Here, differing trends of both parameters versus the concentration of the fluid is seen. The concentration of the fluid is on waning with amplified Brownian motion. Divergent comportment is viewed in Fig. 12. It is owing to the truth that particles are being driven by the Brownian forces in a direction opposite to the concentration gradient that tends the nanofluid more homogeneous. To visualize the influence of the reaction rate constant $R_{c}$ on the concentration profile Fig. 13 is graphed. It is comprehended that concentration is a deteriorated for large rate constant values. Large estimates of $R_{c}$ diminished concentration profile that ultimately strengthen the destructive chemical reaction. Figure 14 is illustrated to look at the impression of the melting heat parameter $M_{a}$ on the temperature profile. It is gathered from the graph that temperature profile is diminished for high growing values of $M_{a} .$ The logic supporting this trend is that higher values of $M_{a}$ results in transferring more heat to the surface from the heated fluid thus decrement in the fluid temperature is witnessed. The effect of temperature difference parameter, diffusion variable, and Brinkman number on entropy optimization are given in Figures 15, 16 and 17. The temperature ratio parameter diminishes the entropy profile while for Brinkman number and diffusion variable entropy generation profile enhances.

Table 2 symbolizes the numerically calculated Nusselt number for varied estimates of Curie temperature, Dimensionless distance, and melting heat parameter. It is witnessed that the Nusselt number is on the decline for growing estimates of all parameters. Table 3 signifies the impact of numerous parameters Schmidt number, the reaction rate constant, and the Brownian motion parameter. It is comprehended that the rate of concentration upsurges for rising estimates of Schmidt number, the reaction rate constant, and the Brownian motion parameter. To corroborate the presented results Table 4 is erected to compare the varied estimates of Prandtl number in limiting case with Chen ^61^ and Abel et al. ^62^ by suppressing the extra parameters. An outstanding association between the results is found.

**Table 2:** Numerically calculated values of Nusselt number.

$\varepsilon$	$\alpha$	$M_{a}$	$Re_{x}^{ - 1/2} Nu_{x}$
0.1	0.5	0.1	1.79373
0.3			1.64217
0.5			1.47769
0.2	0.1		1.17210
	0.2		1.00430
	0.3		0.88453
	0.5		1.79037
			1.75368
			1.71956
		0.2	1.34590
		0.3	1.16070
		0.4	1.09870

**Table 3:** Numerically calculated values of Sherwood number.

$S_{c}$	$R_{c}$	$N_{b}$	$- g^{\prime}(0)$
1.0	0.1	0.1	1.27040
1.5			1.58550
2.0			1.85500
	0.2		1.87980
	0.3		1.90410
	0.4		1.92800
			1.73950
			1.62410
			1.50860
		0.2	1.88660
		0.3	1.91720
		0.4	1.94710

**Table 4:** Comparison of heat transfer rates for varied estimates of Prandtl number $(\Pr )$ with Chen ^61^ and Abel et al. ^62^ by suppressing the extra parameters.

$\Pr$	Chen ^61^	Abel et al. ^62^	Present
0.72	1.0885	1.0885	1.088497
1	1.3333	1.3333	1.333296
3	2.5097	–	2.509689
10	4.7968	4.7968	4.796794

## Concluding remarks

In this exploration, we have examined the flow of ferromagnetic Oldroyd-B nanofluid under the impact of the magnetic field induced by the magnetic dipole. Activation energy amalgamated with the entropy generation is added to the envisioned fluid model. Cattaneo–Christov heat flux model is betrothed in preference to conventional Fourier law with heat generation effect. The numerical solution of the highly nonlinear system is accomplished. The key obesrvations of the presented model are:

- An opposite trend is seen for the velocity and temperature profiles versus growing estimates of the ferromagnetic parameter.
- It is notarized that the concentration profile shows a diminishing tendency for the Brownian motion parameter and an opposite trend is witnessed for the thermophoretic parameter.
- The temperature ratio parameter lessens the entropy profile whilst for Brinkman number and diffusion variable entropy generation profile enhances.
- It is remarked that fluid temperature drops owing to large thermal relaxation time. This trend necessitates additional time to transfer heat to the nearby particles. That is why fluid temperature is plunged.
- The fluid temperature is diminished for high growing values of the melting heat parameter. Actually, large estimates of melting heat parameter transfer more heat to the surface from the heated fluid. Eventually, decline in the fluid temperature is observed.
- A rise in fluid temperature is observed for the augmented heat generation parameter.

## List of symbols

$u$, $v$: Components of velocity along *x*-and *y-*axis (m/s)
$Q_{0}$: Volumetric rate of heat source
$E$: Dimensionless activation energy
$$\Pr$$: Prandtl number
$D_{T}$: Coefficient of thermophoretic movement (m^2^/s)
$$M_{a}$$: Melting parameter
$$S_{c}$$: Schmidt number
$D_{B}$: Coefficient of Brownian movement (m^2^/s)
$R_{c}$: Reaction rate constant
$D_{c}$: Heat generation parameter
$$N_{t}$$: Thermophoresis variables
$N_{b}$: Brownian movement variable
${\text{Re}}_{x}$: Local Reynolds number
$$c$$: Stretching parameter
$c_{s}$: Heat capacity of the solid surface
$$T$$: Nanofluid temperature (K)
$T_{w}$: Nanofluid temperature near the wall (K)
$T_{c}$: Nanofluid free stream temperature (K)
$$k$$: Thermal conductivity
$k_{r}$: Chemical reaction rate
$E_{a}$: Activation energy variable
$$Br$$: Brinkman number
$N_{G} (\eta )$: Entropy generation number
$S^{\prime\prime\prime}_{gen}$: The local volumetric entropy generation rate
$S^{\prime\prime\prime}_{0}$: The characteristic entropy generation rate
$$x,y$$: Coordinates (m)
$L$: The characteristic length (m)
$$T_{\infty }$$: Ambient temprature (K)
$C_{w}$: Constant Surface concentration (*M*)
$RD$: Radiation prameter (rad)
$$\,u_{w}$$: Stretching coefficient
$$f,g$$: Velocity profiles (m/s)
$$\theta_{1} ,\theta_{2} \,$$: Fluid temprature
$$q_{{_{m} \,\,\,}}$$: Heat flux (W/m^2^

## Greek symbols

$\gamma^{*}$: Dimensionless thermal relaxation time
$\mu_{f}$: Viscosity (kg/ms)
$q_{{_{n} \,\,\,}}$: Mass flux (kg/s)
$k_{f}$: Thermal conductivity (WK^−1^ m^−1^)
$\alpha_{2}$: Dimensionless concentration difference
$$C_{p}$$: Specific heat (m^2^/s^2^)
$\lambda$: Viscous dissipation factor
$$\varepsilon$$: Curie temperature
$$\alpha$$: Dimensionless distance
$\tau$: Heat capacity ratio
$\rho_{f}$: Density of the fluid (kg/m^−3^)
$\alpha_{1}$: Dimensionless temperature difference
$\beta$: Ferrohydrodynamic interaction parameter
$\beta_{1} ,\beta_{2}$: Deborah numbers or dimensionless material parameters
$\lambda_{1} ,\lambda_{2}$: Relaxation, retardation times or material parameters
$$\lambda_{0}$$: Magnetic permeability (N/A^2^)
$\alpha_{f}$: Thermal diffusivity of nanofluid (m^2^/s^−1^)
$\lambda^{*}$: Latent heat of the fluid
$\lambda_{3}$: Dimensional thermal relaxation time
$$\eta ,\xi$$: Space variable
$$\upsilon_{f}$$: Kinematic viscosity (m^2^/s)
_K_: Gyromagnetic coefficient
*H*: Elements of the magnetic
$\gamma$: Strength of the magnetic dipole (C m)
$\Phi$: Magnetic dipole scalar potential
*M*: Magnetic effect (Wb/m^2^)
$$\,\,C\,\,$$: Fluid concentration (*M*)
$$C_{c}$$: Constant concentration (*M*)
$$\upsilon$$: Kinematic vescosity (m^2^/s)
$$a\,$$: Constant parameter
$$E_{c}$$: Eckert number
$$Nu_{x}$$: Nusselt number
$$Sh_{x}$$: Sherwood number
